# Semi-automatic detection of myocardial trabeculation using cardiovascular magnetic resonance: correlation with histology and reproducibility in a mouse model of non-compaction

**DOI:** 10.1186/s12968-018-0489-0

**Published:** 2018-10-25

**Authors:** Julien Frandon, Stéphanie Bricq, Zakarya Bentatou, Laetitia Marcadet, Pierre Antoine Barral, Mathieu Finas, Daniel Fagret, Frank Kober, Gilbert Habib, Monique Bernard, Alain Lalande, Lucile Miquerol, Alexis Jacquier

**Affiliations:** 1 Aix-Marseille University, CNRS, CRMBM, Marseille, France; 2 Department of Radiology, Timone University Hospital, Marseille, France; 3 Department of Radiology, Nîmes University Hospital, Nîmes, France; 4 Le2i, Université de Bourgogne Franche-Comté, Dijon, France; 5 CNRS UMR 7288, Developmental Biology Institute of Marseille, Aix-Marseille University, Marseille, France; 6 INSERM, U1039, Radiopharmaceutiques Biocliniques, Université Grenoble Alpes, Grenoble, France; 7 Department of Cardiology, APHM, la Timone Hospital, Marseille, France; 8 Department of MRI, University Hospital Francois Mitterrand, Dijon, France

**Keywords:** Left ventricular non-compaction, Semi-automatic segmentation, CMR, Genetic mouse model, CMR

## Abstract

**Background:**

The definition of left ventricular (LV) non-compaction is controversial, and discriminating between normal and excessive LV trabeculation remains challenging. Our goal was to quantify LV trabeculation on cardiovascular magnetic resonance (CMR) images in a genetic mouse model of non-compaction using a dedicated semi-automatic software package and to compare our results to the histology used as a gold standard.

**Methods:**

Adult mice with ventricular non-compaction were generated by conditional trabecular deletion of *Nkx2–5*. Thirteen mice (5 controls, 8 Nkx2–5 mutants) were included in the study. Cine CMR series were acquired in the mid LV short axis plane (resolution 0.086 × 0.086x1mm^3^) (11.75 T). In a sub set of 6 mice, 5 to 7 cine CMR were acquired in LV short axis to cover the whole LV with a lower resolution (0.172 × 0.172x1mm3). We used semi-automatic software to quantify the compacted mass (M_c_), the trabeculated mass (M_t_) and the percentage of trabeculation (M_t_/M_c_) on all cine acquisitions_._ After CMR all hearts were sliced along the short axis and stained with eosin, and histological LV contouring was performed manually, blinded from the CMR results, and M_t_, M_c_ and M_t_/M_c_ were quantified. Intra and interobserver reproducibility was evaluated by computing the intra class correlation coefficient (ICC).

**Results:**

Whole heart acquisition showed no statistical significant difference between trabeculation measured at the basal, midventricular and apical parts of the LV. On the mid-LV cine CMR slice, the median M_t_ was 0.92 mg (range 0.07–2.56 mg), M_c_ was 12.24 mg (9.58–17.51 mg), M_t_/M_c_ was 6.74% (0.66–17.33%). There was a strong correlation between CMR and the histology for M_t_, M_c_ and M_t_/ M_c_ with respectively: r^2^ = 0.94 (*p < 0.001*), r^2^ = 0.91 (*p < 0.001*), r^2^ = 0.83 (*p < 0.001*). Intra- and interobserver reproducibility was 0.97 and 0.8 for M_t_; 0.98 and 0.97 for M_c_; 0.96 and 0.72 for M_t_/M_c_, respectively and significantly more trabeculation was observed in the M_c_ Mutant mice than the controls.

**Conclusion:**

The proposed semi-automatic quantification software is accurate in comparison to the histology and reproducible in evaluating M_c_, M_t_ and M_t_/ M_c_ on cine CMR.

## Background

Left ventricular (LV) non-compaction (LVNC) is suspected on echocardiography or cardiovascular magnetic resonance (CMR) when a characteristic double-layered aspect of the myocardium with a thick, non-compacted endocardial layer, prominent trabeculations and deep recesses are observed [1–3]. Untreated, LVNC can lead to heart failure, cardioembolic events, and tachyarythmia; it may occur as an isolated cardiomyopathy or in association with other congenital heart diseases [4]*.* Quantification of the total amount of LV trabeculation on CMR has been proposed as a method to diagnose LVNC [3, 5], but manual contouring of all LV trabeculation and compacted LV is time consuming. Recently, Bricq et al. developed a semi-automatic software package to calculate the non-compacted mass (M_t_) that suppresses blood from the trabeculae and evaluate the total amount of LV trabeculation as well as LV mass [6]. The feasibility of this trabeculation quantification algorithm was illustrated in a small group of mice along with their histology data, but no assessment of reproducibility or validation in an animal group of sufficient size was provided.

In this study, the quantification software was compared with histology and tested for accuracy and reproducibility in a mouse model of non-compaction. *Nkx2–5* is a homeodomain transcription factor, which drives the early stages of cardiac morphogenesis*.* Global *Nkx2–5* knock-out in mice causes early embryonic lethality with delayed cardiac development [7, 8]*,* though heterozygous *Nkx2–5* knock-out mice present a spectrum of cardiac anomalies including signs of ventricular non-compaction and cardiac conduction system defects [8–11]. A previous animal study has shown that inducible ventricular knock-out of *Nkx2–5* generates adult mice with an LVNC phenotype [12]. Mutations of *Nkx2–5* have been identified in numerous patients with congenital heart diseases, and some were associated with LVNC [13]. Our goal was to quantify LV trabeculation on CMR in a genetic mouse model of non-compaction using a dedicated semi-automatic software package and to compare our results to the histology used as a gold standard.

## Methods

### Conditional trabecular *Nkx2–5* knock-out mouse model (Fig. 1)

The animal procedures were approved by the University’s ethics committee for animal experimentation (n°40–10,102,012). Animal care was given in compliance with the national and institutional guidelines and this study was performed under the authorization of the local ethical committee. Mice were part of the project “mouse cardiovascular development system” (n° 01055.02). During embryonic development, Connexin-40 (Cx40) is expressed in the trabeculae, therefore we crossed *Nkx2–5* floxed mice [11] with Cx40^cre/+^ mice to allow Cre-inducible deletion of the floxed gene specifically in the trabeculae [14]*.* Cre recombinase mediated-deletion of the *Nkx2–5-Flox gene* was induced with tamoxifen (20 mg/ml, Sigma-Aldrich, St. Louis, Missouri, USA) injected intraperitoneally [15] into pregnant mice (200 μL) for 2 consecutive days or once subcutaneously at 1 day postnatally (P1) (20 μL). The first day of gestation was defined as the morning on which the vaginal plug was found. Thirteen mice were analyzed in 2 groups *(flow chart)*: 5 controls *Nkx2–5*^*+/+*^*;Cx40*^*cre/+*^, and 8 mutant mice: 3 heterozygous *Nkx2–5*^*+/−*^*;Cx40*^*cre/+*^ [9], and 5 homozygous *Nkx2–5*^*flox/−*^*;Cx40*^*cre/+*^ at different stages (4 injected at embryonic stages, and 1 at P1). All mice were sacrificed after CMR and whole hearts were dissected and sectioned in the transversal axis using a cryostat. The eosin stained slices were incubated for 5 min in a 5% aqueous solution (Sigma-Aldrich).

**Fig. 1:**
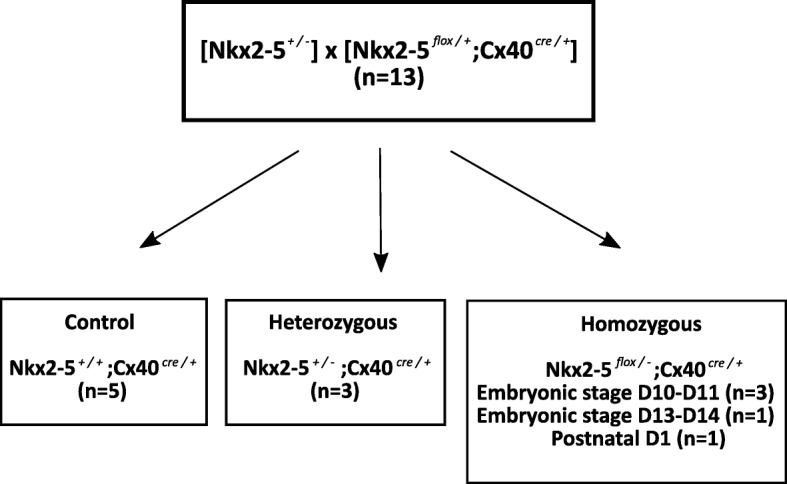
Flow chart

### CMR

CMR scans were performed on adult mice when they weighed 20–30 g, i.e. after 2 to 4 months depending on the strains, using a preclinical 11.75 T system (AVANCE 500 MHz/89 mm wide bore vertical imager (11.75 Bruker Biospin GmbH, Ettlingen, Germany) and a 30 mm diameter transmit-receive radiofrequency resonator. Anesthesia was maintained during CMR with 1–2% isoflurane in a constant flow of ambient air (270 ml/min) through a nose cone using a dedicated vaporizer (Univentor anaesthesia unit, Univentor high precision instruments, Zejtun, Malta). Body temperature was maintained at 37 °C. Respiration rate and heart rate were monitored and the signals were used to trigger the CMR acquisitions using a monitoring and gating system (SA Instruments, Inc. Stony Brook, New York, USA). High resolution spoiled gradient echo cine CMR (repetition time 15 ms, echo time 1.68 ms, flip angle 30°, slice thickness 1 mm, in-plane resolution 0.086 × 0.086 mm^2^) was performed in short axis view, at mid base-apex level.

In a subset of 6 mice (1 control, 5 mutant), whole heart spoiled gradient echo cine CMR with lower spatial resolution was added to the high resolution acquisition (repetition time 5 ms, echo time 1.51 ms, flip angle 20°, slice thickness 1 mm, in-plane resolution 0.172 × 0.172 mm^2^) in short axis view. Five to seven slices were acquired from base to apex to cover the whole left ventricle. The end-diastolic frames were segmented with the software to assess the extent of the trabeculation across LV.

### Image analysis

**Fig. 2:**
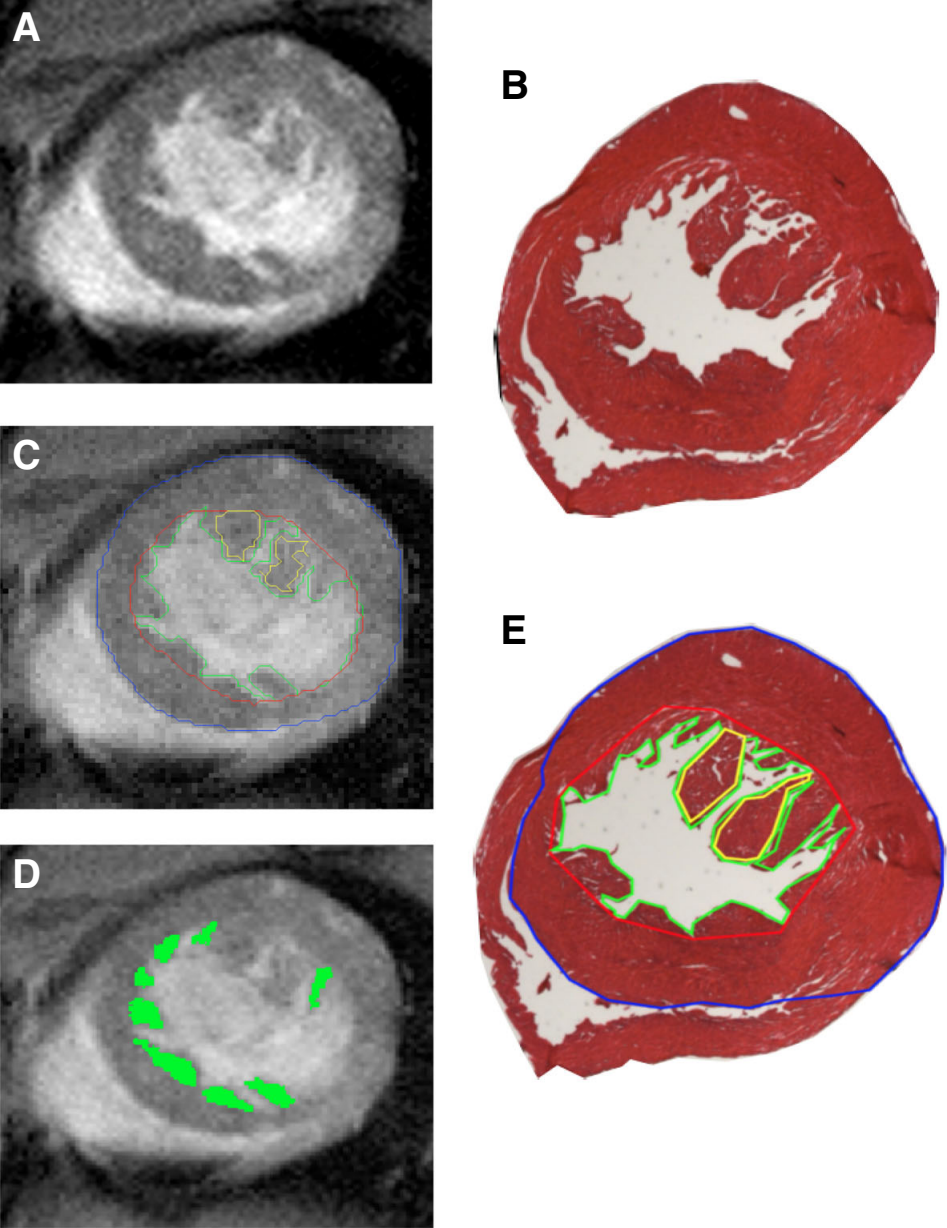
Example of left ventricular (LV) high resolution CMR segmentation and corresponding eosin stained histological images. **a**-**b**: Example of a midventricular short axis, cine image from a mutant mouse heart (**a**) and slice at the same level from eosin stained histological slide (**b**). **c**-**d**: Semi-automatic LV segmentation of the cine image: the epicardial border appears in blue, the endocardial border in red, the trabecular border in green (**c**) and the trabecular surface in green (**d**). The papillary muscles were segmented and included in the compacted area (yellow line) (**c**). **e**: Manual LV segmentation of the histological slice using the same segmentation procedure: epicardial border in blue, endocardial border in red, trabecular border in green. The papillary muscles were segmented and included in the compacted area (yellow line)

For the evaluation of LV trabeculation, the end diastolic frame from the cine imaging was used to segment the myocardium with the dedicated software previously described [6]. The method requires a seed indicated by the user to initialize the selected slice. The non-compacted region was obtained using this seed and a region-growing algorithm. This non-compacted contour was then used to initialize an active contour to determine the endocardial and epicardial borders. When necessary, each contour was manually corrected. The papillary muscles were then segmented using a semi-automatic threshold tool based on the difference between bright blood and darker myocardial intensities. Afterwards, blood was removed from the trabeculae using the same thresholding tool. The trabeculae were defined at the end-diastolic phase as dark myocardium protruding from the LV wall into the bright LV cavity. Papillary muscles were included in the myocardial mass. If the papillary muscles could not be clearly distinguished from the trabeculation on the short-axis images, they were treated as trabeculation (Fig. 2) [6].

The trabeculated area (M_t_) was defined as the area including all LV trabeculations excluding the papillary muscles and LV blood between the trabeculae. Compacted area (M_c_) was defined by the myocardial area including the papillary muscles and excluding LV trabeculation. The knowledge of the slice thickness of 1 mm allows the calculation of the trabeculated and compacted mass in each slice. Percentage of LV trabeculation (M_t_/M_c_) was expressed as a percentage of the trabeculated area computed by M_t_/M_c_ × 100.

To investigate intraobserver variability, observer 1 (J.F., 5 years of experience) repeated the measurements on 10 cine images at least 1 month after the first examination to minimize recall bias. A second independent observer (Z.B., 2 years of experience) repeated the different measurements on the same acquisitions to assess interobserver variability.

All mice were sacrificed after CMR and whole hearts were dissected and sectioned in the transversal axis using a cryostat. Cryosections were 20 μm thick. From these cryosections, we visually selected the one that best corresponded to the CMR slice level. Papillary muscle shape and size and trabeculation distribution were selected as land marks to perform a visual co-registration between CMR and the histology. In the subset of 6 mice, 5 to 7 histological sections were selected per animal to be consistent with the 5 to 7 CMR stacks acquired from base to apex using the same co-registration method.

Each histological slice was analyzed by observer 1 (J.F.) who was blinded to the CMR results. The segmentation was done manually with Image J (version 1.49 v, National Institute of Health, Bethesda, Maryland, USA) using the same segmentation procedure (Fig. 2).

### Statistical analysis

All mouse group characteristics are reported as median values and range. The values were compared between the groups using Kruskal-Wallis and Mann Whitney U tests. The results were considered as significant when *p* < .05. Linear regression and Pearson correlation coefficients between the histology and CMR were assessed. The histological and CMR results for M_c_, M_t_ and M_t_/M_c_ were compared using Bland-Altman analysis and taking the histological data as a reference. Interobserver and intraobserver reproducibilities were assessed by computing the intraclass correlation coefficient (ICC) with a confidence interval (CI) of 95% using Bland-Altman analysis.

## Results

### Mice characteristics

There were no significant differences between the control and mutant groups in terms of age, body weight or heart weight (Table 1). CMR was feasible with a good quality of image in all mice. A total of 13 cine images were evaluated. CMR was performed on adult mice on median day 63 (range: 47–120 days). Median body and heart weight at the time of CMR were 26.5 g (range 16.5–34.0 g) and 185 mg (range 124–233 mg), respectively. Histological staining was successful in all cases.

**Table 1:** Mice characteristics at time of CMR: median values (min-max)

	Control (*n* = 5)	Mutant (*n* = 8)	*P value*
Age at time of CMR (days)	118 (60–120)	60 (47–112)	0.17
Total weight (g)	30.6 (24–33)	24 (16.5–34)	0.30
Heart weight (mg)	132 (124–181)	196 (153–233)	0.07

### Study of trabeculation across the whole LV

**Fig. 3:**
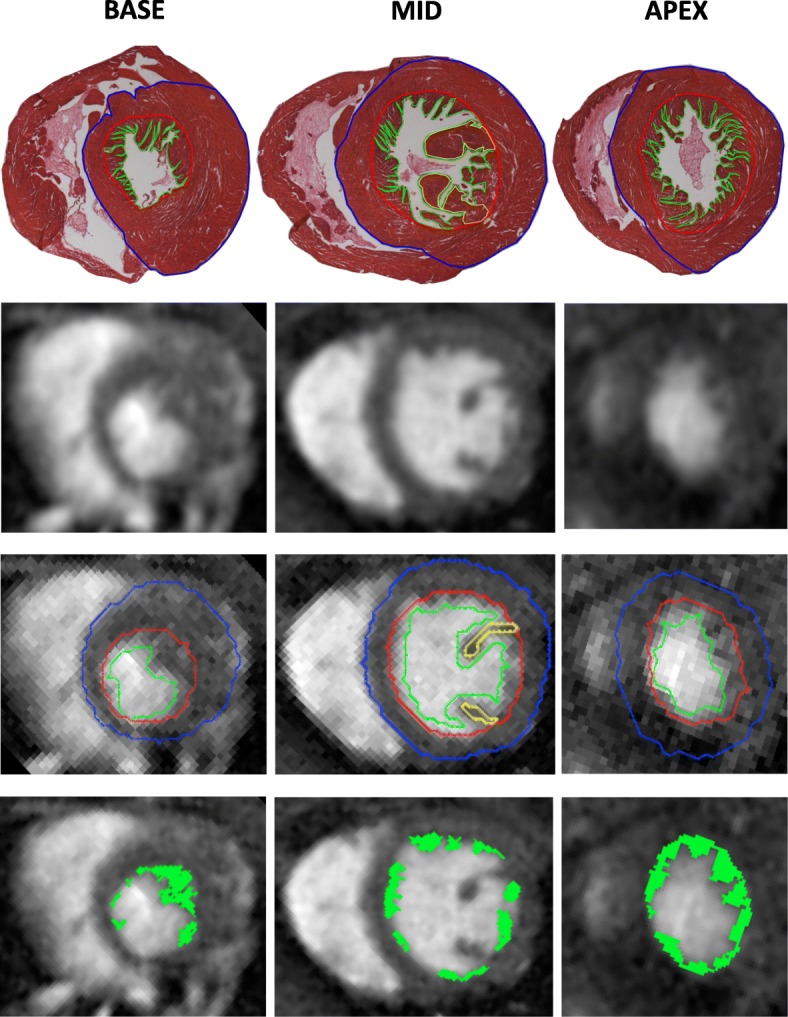
Example of left ventricular whole heart CMR segmentation and corresponding eosin stained histological images. Example of a basal (left), midventricular (middle) and apical (right), histological slices with the corresponding short axis cine image from a mutant mouse heart. The epicardial borders appear in blue, the endocardial borders in red, the papillary muscles in yellow, the trabecular borders in green and the trabecular surface after manual deletion of blood in green (bottom line)

Processing took 15 ± 0.5 min for one whole heart examination, including computation time and user interaction. Automatic epicardial, endocardial and trabeculation border detection took less than 1 min per slice. Semi automatic papillary muscles detection took less than 30 s, whereas around 1.5 min were required to segment blood within the trabeculation net per slice (Fig. 3).

The results of whole heart segmentation for M_t_, M_c_ and M_t_/M_c_ are summarized in Table 2. There was no statistical difference between basal, midventricular and apical parts for M_t_ (*p = 0.61*), M_c_ (*p = 0.48*) and M_t_/M_c_ (*p = 0.35*).

**Table 2:** Trabeculae segmentation: whole heart CMR vs histology

	CMR	Histology	*P value*
Whole heart M_t_ (mg) median values (min–max)	6.31 (4.76–10.22)	4.68 (2.3–8.4)	0.16
Apical M_t_ (mg)	2.68 (1.56–4)	1.68 (0.6–2.93)	0.86
Midventricular M_t_ (mg)	2.59 (1.57–5)	1.75 (0.65–2.47	0.94
Basal M_t_ (mg)	2.28 (1.21–5)	0.97 (0.36–2.06)	0.41
Whole heart M_c_ (mg)	57.52 (36.72–90.8)	52.13 (34.63–86.59)	0.7
Apical M_c_ (mg)	17.07 (8.87–27)	17.13 (12.26–25.97)	0.89
Midventricular M_c_ (mg)	24.1 (15.73–30)	21.7 (16.09–32.34)	0.56
Basal M_c_ (mg)	19.42 (12.1–29.5)	17.95 (7.12–28.28)	0.73
Whole heart M_t_/M_c_(%)	12.95 (6.36–17.57)	9.19 (3.12–16)	0.75
Apical M_t_/M_c_ (%)	15.75 (7.61–33.03)	10.85 (7.15–23.9)	0.9
Midventricular M_t_/M_c_ (%)	10.59 (6.9–16.67)	8.51 (2.51–12.02)	0.73
Basal M_t_/M_c_ (%)	9.12 (7.99–14.67)	5.4 (3.66–7.67)	0.44

The results of the Bland-Altman test performed with the histological data as reference showed good agreement with a subtle overestimation of myocardial mass compared to the histology: + 1.89 ± 1.08 mg for M_c_, + 4.26 ± 2 mg for M_c_ and + 2.35 ± 1.83% for M_t_/M_c_. There was a strong correlation between the histology and the whole heart cine imaging for M_c_: r^2^ = 0.98 (*p < 0.001*); and good for M_t_: r^2^ = 0.74 (*p = 0.017*); and M_t_/M_c_: r^2^ = 0.81 (*p = 0.009*).

### Trabecular segmentation and histological correlation on high resolution CMR

**Fig. 4:**
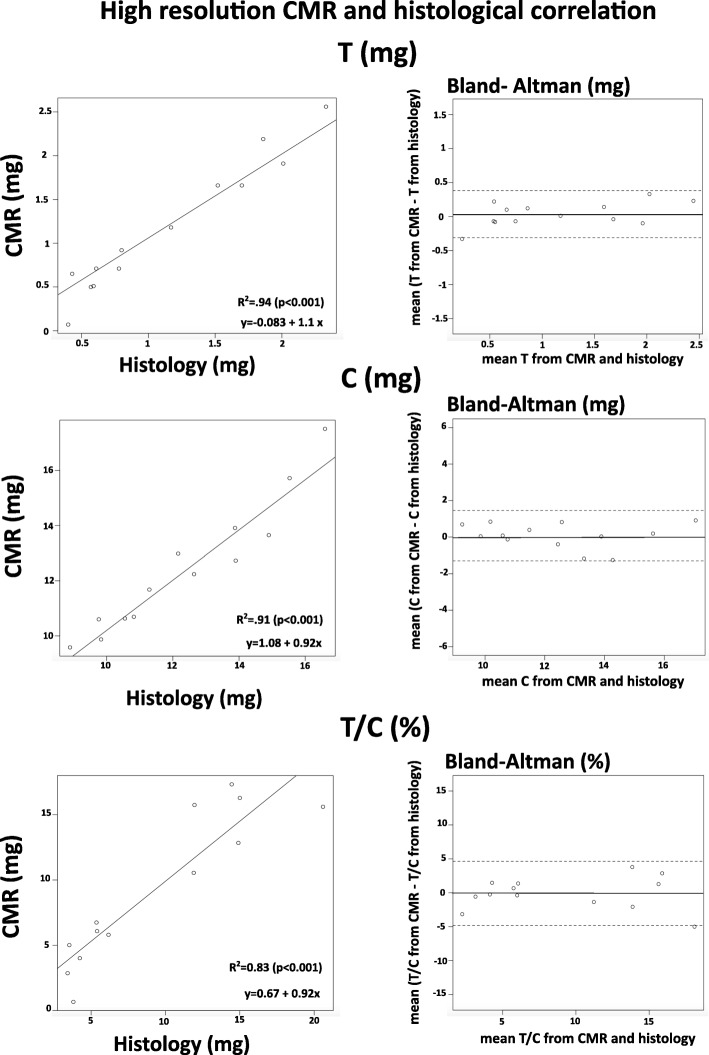
Correlation between high resolution CMR and histology. Linear regression (left) showing the relationship between histology and CMR quantifications of M_t_ (T) (top), M_c_ (C) (middle) and M_t_/M_c_ (T/C) (bottom). Bland-Altman plots (right) showing the agreement between histology and CMR quantifications of T (top), C (middle) and T/C (bottom)

Segmentation was feasible in all mice (Table 3). The median value of M_t_ in all mice by CMR showed a high agreement with histology (0.92 mg CMR vs 0.8 mg histology, r^2^ = 0.94, *p* < 0.001). The median value of M_c_ in all mice by CMR showed a high agreement with histology (12.24 mg CMR vs 12.16 mg histology, r^2^ = 0.91, *p* < 0.001). The median value of M_t_/M_c_in all mice by CMR showed a good agreement with histology (6.74% CMR vs 6.17% histology, r^2^ = 0.83, *p* < 0.001, Table 3, Fig. 4). The results of the Bland-Altman test using the histological data as a reference showed high agreement (Fig. 4).

**Table 3:** Trabeculae segmentation: high resolution CMR vs histology

	CMR	Histology	*P value*
M_t_ (mg) median values (min–max)	0.92 (0.07–2.56)	0.8 (0.4–2.23)	0.88
M_c_ (mg)	12.24 (9.58–17.51)	12.16 (8.89–16.6)	0.74
M_t_/M_c_ (%)	6.74 (0.66–17.33)	6.17 (3.42–14.45)	0.83

#### Intra- and interobserver reproducibility

**Fig. 5:**
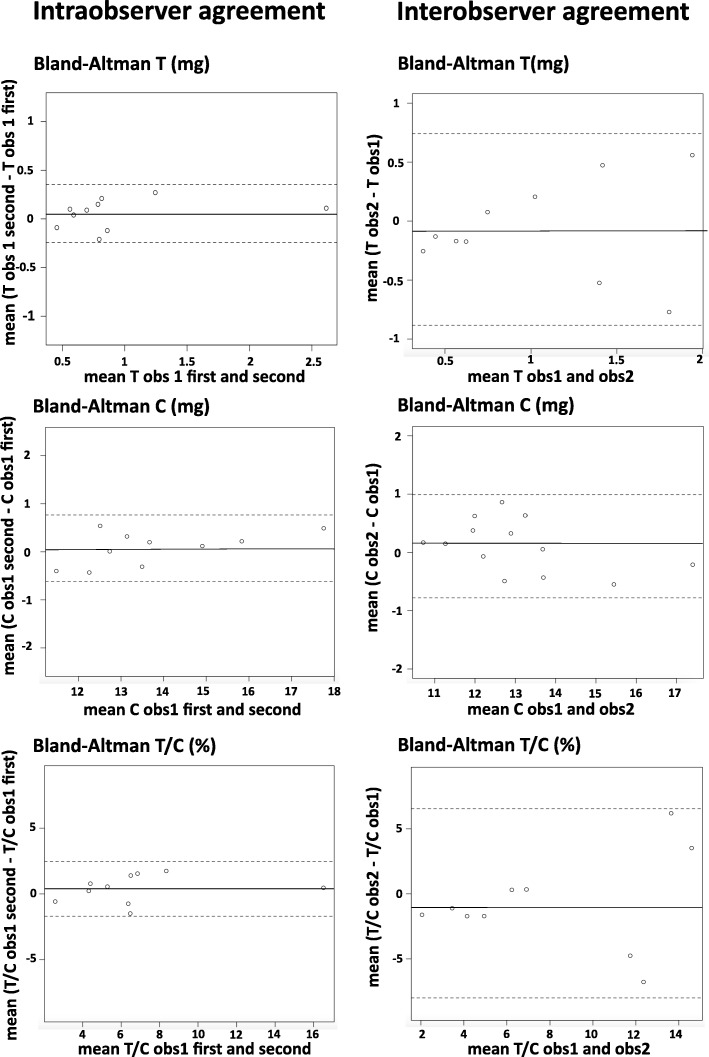
Intra- and Interobserver agreement. Bland and Altman plots showing the agreement for intraobserver measurements (left), and interobserver measurements (right) for CMR quantification of M_c_ (top), M_t_ (middle), M_t_/M_c_ (bottom). Observer 1 first measurements were chosen as reference

Intraobserver reproducibility assessed by ICC was high for M_t_, M_c_ and M_t_/M_c_ with 0.97 (0.6–0.99), 0.98 (0.94–0.99) and 0.93 (0.84–0.97), respectively (Fig. 5). Interobserver reproducibility was high for M_c_: 0.97 (0.88–0.99) and moderate for M_t_ and M_t_/M_c_: 0.80 (0.55–0.85), 0.72 (0.49–0.85), respectively. The results of the Bland-Altman analysis comparing the 2 observers showed very good agreement with a mean difference close to 0: 0.01 ± 0.54 mg (coefficient of variation: ± 33.23%) for M_t_, 0.63 ± 0.45 mg (± 7.63%) for M_c_ and − 0.56 ± 4.14% (± 37.69%) for M_t_/M_c_.

### Trabecular segmentation and mutant mice on high resolution CMR

**Fig. 6:**
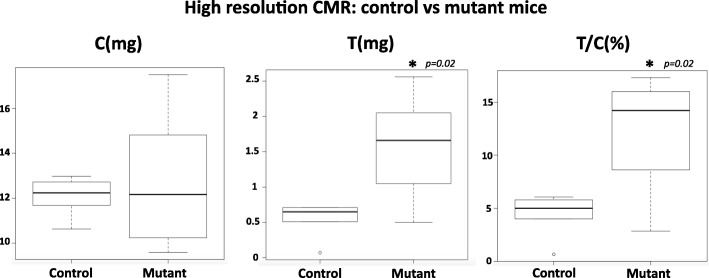
comparison between control and mutant mice on high resolution CMR. Box-and-whisker plots comparing M_c_ (left), M_t_ (middle) and M_t_/M_c_ (right) quantification on CMR, between control and mutant mice

The M_c_ part of the myocardium from the CMR mid-LV cine images showed no significant difference when the mutant mice: median 12.17 mg (range 9.58–17.51 mg) were compared to the control mice 12.24 mg (range 10.63–12.98 mg) (*p = 0.94*) (Table 4). The M_t_ and M_t_/M_c_ calculated using CMR were significantly higher in the mutants in comparison to the control mice with 1.66 mg (range 0.5–2.56 mg) vs 0.65 mg (range 0.07–0.71 mg) (*p = 0.02*) for M_t_; and 14.22% (range 2.86–17.33 mg) vs 5% (range 0.66–6.08%) (*p = 0.02*) for M_t_/M_c_ (Fig. 6).

**Table 4:** Trabecular segmentation on high resolution CMR: control vs mutant mice

	Control (n = 5)	Mutant (n = 8)	*P value*
M_t_ from histology (mg) median values (min–max)	0.59 (0.04–0.78)	1.61 (0.57–2.33)	**0.01**
M_t_ from CMR (mg)	0.65 (0.07–0.71)	1.66 (0.5–2.56)	**0.02**
M_c_ from histology (mg)	12.16 (10.55–13.91)	12.35 (8.89–15.53)	1.00
M_c_ from CMR (mg)	12.24 (10.63–12.98)	12.17 (9.58–17.51)	0.94
M_t_/M_c_ from histology (%)	4.24 (3.54–6.17)	13.72 (3.43–19.12)	0.06
M_t_/M_c_ from CMR (%)	5.01 (0.66–6.08)	14.21 (2.86–17.33)	**0.02**

*p* < .05 indicate entries in boldface

## Discussion

In this murine CMR study, we assessed the performance of a semi-automatic software program dedicated to the quantification of compacted, trabeculated mass and found a high correlation between the histology and CMR. The software also showed a low intra- and intrerobserver variability.

A few earlier studies assessed trabeculation in mice using different methods. Kosaka et al. assessed trabeculation by measuring the M_t_ area [16], however, their histological validation was performed on sliced hearts in 4-chamber views. Yang et al. and Chen et al. also studied trabeculation in embryonic mice hearts [17, 18] by measuring M_t_ thickness on histological pseudo 4-chamber slices. These data are difficult to compare with our results.

In our study, the LV of mice seemed to be less trabeculated compared to human LV with a more homogeneous distribution of trabeculation over the entire LV. In humans there is a basal to apical gradient with increase of trabeculation from base to apex [6, 19]. In mice, the apical and midventricular part of the LV seemed to be hypertrabeculated. We chose a midventricular section for high resolution cine imaging to facilitate co registration with the histology using the papillary muscles as landmarks. A comparison between CMR and the histology was feasible in all mice and the correlation was high, however, the correlation with the histology was slightly lower than for the compacted part due to the small size and amount of trabeculation, partial volume effects and flow artifacts.

The software we used for this purpose quantifies trabeculation for the whole LV and requires just one click in the LV cavity to initialize the region-growing algorithm to detect the non-compacted borders. We used interactive thresholding to segment the papillary muscles and to extract blood from the non-compacted area. The software provided an accurate, reproducible, non-invasive semi-automatic calculation of LV compacted and trabeculated mass in adult mice on the cine imaging. We were able to show a significant increase of LV trabeculation in mice with conditional trabecular deletion of the Nkx2.5 gene compared to control mice. The semi-automatic software was capable of differentiating between control and mutant mice. Assessing the correlation between the amounts of trabeculae and clinical phenotypes may contribute to a better understanding of hypertrabeculation in humans.

The reproducibility for M_c_ was high as shown earlier by Bricq et al. in their evaluation of normal trabeculation in humans (60 volunteers) [6]. Interobserver reproducibility was moderate for M_t_ and M_t_/M_c_ mainly due to additive systematic measurement errors between M_t_ and M_c_. This could be explained by the difference of segmentation due to partial volume around trabecules between the two observers. Intraobserver reproducibility remained very high for M_t_, M_c_, and M_t_/M_c_; this emphasizes the necessity for a strict method of segmentation, notably for trabeculation. Measurement variation for M_t_ and M_t_/M_c_ could be clinically significant for small amounts of trabeculae.

The quantification of trabeculated mass has previously been used in several human studies assessing hypertrabeculation [3, 5, 6]. The global trabeculation ratio of 6.74% was low compared to human reference even for mutant mice with a T/C of 14.22%. In their study on 60 healthy subjects, Briq et al. found a M_t_/M_c_ratio of 10.26% with more trabeculation in the apex and lateral parts [6]. In human studies, the diagnosis of LVNC is still a matter of debate and there is no standard of reference for LV trabeculation quantification. Petersen et al. measured the M_t_/M_c_thickness on human subjects at the point where trabeculation was the most pronounced [2]. A threshold M_t_/M_c_ thickness of 2.3 has been proposed to diagnose non-compaction [2], however Kawel et al. have shown that 43% healthy subjects from the Multiethnic Study of Atherosclerosis (MESA) Study have a M_t_/M_c_ > 2.3 [19]. In this study, the evaluation was based on area segmentation and mass calculation. We arbitrarily selected a mid-LV slice to compare with the histological data. Captur et al. characterized the trabeculae differently, using a parameter correlated with LV endocardial complexity to calculate a fractal index that they applied to embryonic mice hearts by using high-resolution microscopy [20–22]. Hypertrabeculation is also a phenotype that could be encountered in several other cardiomyopathies such as hypertrophic cardiomopathy. Furthermore some first-degree relatives of patients with LVNC may express a hypertrabeculated phenotype. An accurate tool to quantify LV trabeculation could prove to be useful to define the phenotype of the patient more precisely.

The principal limitation of the study is the fact that our software is not fully automatic and this next step would require further resources such as artificial intelligence tools to allow completely automated segmentation of LV trabeculation. The co-registration between CMR and histology was carried out visually. Despite the care and accuracy we put into that step a misregistration bias may have been included.

Furthermore, we quantify trabeculation as a mass. This is an approximation of the truth due to the partial volume effect. M_t_seen on CMR may not always extend through the entire thickness of the slice. It is more likely that trabeculation represents a partial volume through the slice thickness. What we express as M_t_is thus some uncertain value between blood and myocardium. To be consistent with the literature we have used the term trabeculated mass.

The method requires assessment in clinical practice to define cut off values for normal LV trabeculation and excess trabeculation.

## Conclusions

The proposed post-processing software allowed accurate and reproducible quantification of the amount of LV trabeculation in mice from CMR. In comparison to the histology, the semi-automatic software was accurate in its evaluation of M_c_, M_t_ and M_t_/M_c_. Trabeculation seems to increase dramatically when the deletion of the *Nkx2.5* gene is induced at different embryonic development stages.

### Clinical competences

This study assesses the accuracy and reproducibility of a new software program to evaluate left ventricular T. The semi-automatic software shows a high degree of accuracy and reproducibility compared to the histology as a reference. This software program could be used in humans to quantify the total amount of trabeculation.

### Translational outlook

We showed that it was possible to assess trabeculation in a non-invasive manner and that we could differentiate mutant from control mice. LV non-compaction remains controversial and has no gold standard diagnosis. This semi-automatic software could help to discriminate normal from hypertrabeculated patients due to its accuracy in calculating the amount of LV trabeculation.

## Abbreviations

CI: Confidence interval
CMR: Cardiovascular magnetic resonance
Cx40: Connexin 40
D: Day
ICC: Intra-class correlation coefficient
LV: Left ventricle/left ventricular
LVEF: Left ventricular ejection fraction
LVNC: Left ventricular non-compaction
M_c_: Compacted mass
M_t_/M_c_: Percent of trabeculation computed by M_t_/M_c_ × 100
M_t_: Trabeculated mass
P1: Postnatal day 1
PCR: Polymerase chain reaction
RV: Right ventricle/right ventricular
